# Neonatal amygdala resting-state functional connectivity and socio-emotional development in very preterm children

**DOI:** 10.1093/braincomms/fcac009

**Published:** 2022-01-27

**Authors:** Dana Kanel, Lucy D. Vanes, Gareth Ball, Laila Hadaya, Shona Falconer, Serena J. Counsell, A. David Edwards, Chiara Nosarti

**Affiliations:** Centre for the Developing Brain, School of Imaging Sciences & Biomedical Engineering & Imaging Sciences, King’s College London, London, UK; Department of Child and Adolescent Psychiatry, Institute of Psychiatry, Psychology and Neuroscience, King’s College London, London, UK; Centre for the Developing Brain, School of Imaging Sciences & Biomedical Engineering & Imaging Sciences, King’s College London, London, UK; Department of Child and Adolescent Psychiatry, Institute of Psychiatry, Psychology and Neuroscience, King’s College London, London, UK; Centre for the Developing Brain, School of Imaging Sciences & Biomedical Engineering & Imaging Sciences, King’s College London, London, UK; Developmental Imaging, Murdoch Children’s Research Institute, Melbourne, Australia; Department of Paediatrics, University of Melbourne, Melbourne, Australia; Centre for the Developing Brain, School of Imaging Sciences & Biomedical Engineering & Imaging Sciences, King’s College London, London, UK; Department of Child and Adolescent Psychiatry, Institute of Psychiatry, Psychology and Neuroscience, King’s College London, London, UK; Centre for the Developing Brain, School of Imaging Sciences & Biomedical Engineering & Imaging Sciences, King’s College London, London, UK; Centre for the Developing Brain, School of Imaging Sciences & Biomedical Engineering & Imaging Sciences, King’s College London, London, UK; Centre for the Developing Brain, School of Imaging Sciences & Biomedical Engineering & Imaging Sciences, King’s College London, London, UK; Neonatal Unit, Evelina London Children’s Hospital, London SE1 7EH, UK; MRC Centre for Neurodevelopmental Disorders, King’s College London, UK; Centre for the Developing Brain, School of Imaging Sciences & Biomedical Engineering & Imaging Sciences, King’s College London, London, UK; Department of Child and Adolescent Psychiatry, Institute of Psychiatry, Psychology and Neuroscience, King’s College London, London, UK

**Keywords:** resting-state fMRI, socio-emotional outcomes, very preterm, child development

## Abstract

Very preterm children are more likely to exhibit difficulties in socio-emotional processing than their term-born peers. Emerging socio-emotional problems may be partly due to alterations in limbic system development associated with infants’ early transition to extrauterine life. The amygdala is a key structure in this system and plays a critical role in various aspects of socio-emotional development, including emotion regulation. The current study tested the hypothesis that amygdala resting-state functional connectivity at term-equivalent age would be associated with socio-emotional outcomes in childhood. Participants were 129 very preterm infants (<33 weeks' gestation) who underwent resting-state functional MRI at term and received a neurodevelopmental assessment at 4–7 years (median = 4.64). Using the left and right amygdalae as seed regions, we investigated associations between whole-brain seed-based functional connectivity and three socio-emotional outcome factors which were derived using exploratory factor analysis (*Emotion Moderation*, *Social Function* and *Empathy*), controlling for sex, neonatal sickness, post-menstrual age at scan and social risk. Childhood *Emotion Moderation* scores were significantly associated with neonatal resting-state functional connectivity of the right amygdala with right parahippocampal gyrus and right middle occipital gyrus, as well as with functional connectivity of the left amygdala with the right thalamus. No significant associations were found between amygdalar resting-state functional connectivity and either *Social Function* or *Empathy* scores. The current findings show that amygdalar functional connectivity assessed at term is associated with later socio-emotional outcomes in very preterm children.

## Teaser

This study shows that both stronger and weaker resting-state functional connectivity between the amygdala and cortical and limbic areas in the neonatal period is associated with socio-emotional outcomes in children who were born very preterm.

## Graphical Abstract

**Figure:**
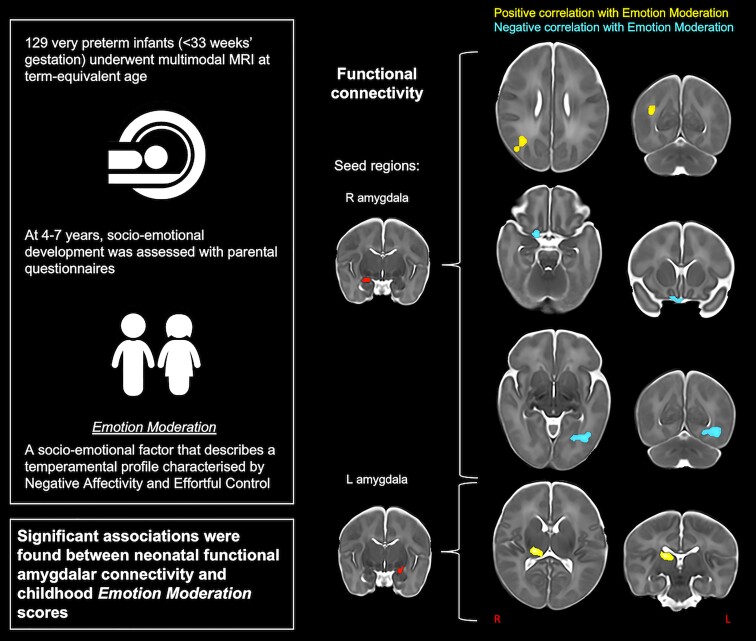

## Introduction

Approximately a quarter of very preterm children (born at <33 weeks’ gestation) experience persisting behavioural difficulties, such as inattention, anxiety, socio-emotional and internalizing problems.^1^ Furthermore, very preterm children have elevated rates of sub-threshold psychiatric symptoms, which may impact their quality of life and the forming of peer relationships.^2^ The presence of sub-threshold symptoms in paediatric settings increases children’s likelihood of developing full psychiatric disorders^3^ and preterm-born youth, aged 10–25 years, were shown to be over 3.5 times more likely to receive a clinical psychiatric diagnosis than their full-term peers.^4^ Recent figures indicated that 21% of preterm children aged 9 met diagnostic criteria for an anxiety disorder, compared with 13% of term-born controls.^5^

However, overt psychiatric symptoms emerge slowly and long after the processes contributing to the psychiatric disorder have begun. Within a conceptual framework suggesting that mental illness lies on a continuum with typical behavioural traits,^6^ longitudinal studies of childhood development can recognize the earliest signs, or even precursors, of mental disorders that only emerge later in life.^7,8^ Within this framework, socio-emotional problems observed in early childhood following very preterm birth, including atypical social development, emotion dysregulation and internalizing problems^9–12^ may represent precursors of later psychopathology.

Behavioural difficulties could result from altered neurodevelopment following very preterm birth, as the immature nervous system is vulnerable to injury.^13^ Very preterm infants and children compared with term-born controls show widespread alterations in structural brain connectivity and network architecture^14–19^ as well as in functional brain connectivity, in terms of both network strength and complexity.^20–25^

Functional connectivity alterations in preterm neonates have been studied in relation to childhood cognitive and behavioural outcomes.^26–28^ Of particular interest with respect to socio-emotional development are the amygdalae, bilateral limbic regions that are central to the brain’s emotional processing networks.^29–32^ Research has highlighted the role of the amygdalae in the development of anxiety,^33^ possibly implicating their connectivity with the prefrontal cortex, which exerts top-down regulation of fear responses.^34,35^ Functional connectivity of the amygdalae at rest (i.e. resting-state functional connectivity, rs-FC) has been associated with internalizing and externalizing difficulties, including anxiety and aggression, in both healthy and clinical cohorts of children and adolescents.^36–40^ Further, altered neonatal amygdalar rs-FC has been shown to predict later socio-emotional outcomes, including the development of negative affect, fear, sadness and emotion regulation.^41–44^

Very preterm children and adolescents exhibit altered structural and functional amygdalar development compared with term-born controls, showing smaller volumes^45^ and reduced connectivity.^46–48^ Using a longitudinal design, a recent study found that rs-FC between the left amygdala and several regions (including the medial prefrontal cortex, posterior cingulate and anterior insula) measured at term-equivalent age (TEA) in very preterm infants predicted internalizing symptoms at 2 years of age.^49^ The identification of neurobiological substrates that are later associated with behavioural difficulties in very preterm children could be used to inform risk stratification within a vulnerable sample with heterogeneous outcomes.

The current longitudinal study aimed to extend previous findings by evaluating associations between neonatal amygdalar rs-FC in very preterm infants and distinct facets of socio-emotional development in early childhood. We recently showed that structural connectivity of the neonatal limbic system [i.e. neonatal diffusion characteristics of the uncinate fasciculus (UF), which connects the amygdalae to the orbitofrontal cortex (OFC)] was related to socio-emotional outcomes in very preterm children.^50^ Building on this finding, the aim of the current study was to investigate—in the same cohort—whether these socio-emotional outcomes would also be related to neonatal limbic functional connectivity. We hypothesized that altered rs-FC of the amygdalae would be associated with poorer childhood socio-emotional outcomes, although a direction of association was not predicted, as previous studies reported both positive and negative correlations between amygdalar rs-FC and mental health outcomes.^49^ Additionally, we explored function–structure associations between neonatal amygdalar rs-FC and the relevant diffusion characteristics of the UF (i.e. fractional anisotropy), which were previously shown to relate to childhood socio-emotional functions.^50^

## Materials and methods

### Participants

Five hundred and eleven infants were originally recruited in 2010–13 as part of the Evaluation of Preterm Imaging study (ePrime, EudraCT 2009-011602-42),^51^ from hospitals within the North and Southwest London Perinatal Network. Inclusion criteria were birth <33 weeks’ gestation and maternal age over 16 years. Exclusion criteria were the presence of major congenital malformation, prior magnetic resonance imaging (MRI), metallic implants, parents unable to speak English or being subject to child protection proceedings. Infants underwent MRI at TEA, defined as 38–44 weeks.

Complete resting-state fMRI (rs-fMRI) data were available for 298 neonatal scans after the removal of incomplete or corrupt data. Infants with post-menstrual age (PMA) at scan ≥45 weeks were excluded, as well as those with major destructive brain lesions, defined as periventricular leucomalacia, haemorrhagic parenchymal infarction and other ischaemic or haemorrhagic lesions,^52^ but not including punctate lesions or diffuse excessive high signal in white matter on T_2_-weighted images.

Two hundred and fifty-one children were invited for a neurodevelopmental follow-up assessment at the Centre for the Developing Brain, St Thomas’ Hospital, London, between the ages of 4 and 7. Complete follow-up behavioural data were available for 151 children. The final sample consisted of 129 very preterm-born participants [mean GA = 29.4 weeks (SD = 2.27)] with neonatal resting-state functional, T_1_- and T_2_-weighted MRI at TEA [mean age at scan = 42.2 weeks (SD = 1.44)] and subsequent childhood follow-up assessment [mean age at assessment = 5.04 years (SD = 0.80)].

Written informed consent was obtained from participants’ carer(s) following procedures approved by the National Research Ethics Committee (14/LO/0677). The study was carried out in accordance with the Code of Ethics of the World Medical Association (Declaration of Helsinki).

### Perinatal socio-demographic and clinical data

Perinatal socio-demographic and clinical data were collected, with permission, from the Standardised Electronic Neonatal Database. Index of multiple deprivation (IMD) score, a proxy for socioeconomic status, was computed from parental postcode at the time of infant birth (Department for Communities and Local Government, 2011; https://tools.npeu.ox.ac.uk/imd/). The IMD measures social risk by comparing each neighbourhood to all others in the country and is based on seven domains of deprivation: income, employment, education skills and training, health and disability, barriers to housing and services, living environment and crime. Maternal education was defined as age upon leaving full-time education, divided into two categories: (i) at or before 19 years and (ii) after 19 years,^53^ as in the UK, this cutoff coincides with the completion of graduate studies.^54^

Clinical data were summarized into a ‘neonatal sickness index’ (please refer to Kanel *et al*.^50^ for further details) which consisted of the following five variables: GA, days on total parenteral nutrition, days on continuous positive airway pressure, days on mechanical ventilation and surfactant administration. Higher values reflected greater clinical risk.

Sample characteristics for the original neonatal sample and follow-up subsamples, with available behavioural and MRI + behavioural data, are shown in Table 1. The current complete sample (Complete (MRI + behavioural) sample, *n = *129) did not differ from the baseline neonatal sample (Baseline (MRI) sample, *n* = 298) in terms of GA, PMA, neonatal sickness index, sex or maternal education. The current complete sample also did not differ from the behavioural follow-up subsample (Follow-up (behavioural) sample, *n = *151) in terms of age at childhood assessment (*t* = −1.221, *P* = 0.231) or full-scale intelligence quotient (IQ) (*t* = 0.124, *P* = 0.902).

**Table 1:** Participants’ socio-demographic characteristics

		Baseline (MRI) sample (*N* = 298)	Follow-up (behavioural) sample (*N* = 151)	Complete (MRI + behavioural) sample (*N* = 129)	Baseline versus complete sample
GA (weeks), median (range)		30.43 (23.57–32.86)	30.14 (24–32.86)	30.3 (24–32.9)	*t* = 0.727, *P* = 0.468
PMA (weeks), mean (SD)		42.12 (1.53)	42.22 (1.42)	42.2 (1.44)	*t* = −0.607, *P* = 0.544
Neonatal sickness index, median (range)		−0.29 (−1.34 to 2.55)	−0.32 (−1.34 to 2.05)	−0.29 (−1.34 to 2.05)	*t* = −0.112, *P* = 0.911
Female (number, %)		146 (49.0%)	69 (45.7%)	61 (47.3%)	*χ* ^2^ = 0.16, *P* = 0.691
Maternal education ≥19 years, number (%)		200 (67.11)	117 (77.5)	95 (73.64)	*χ* ^2^ = 1.57, *P* = 0.210
IMD score quintiles, *n* (%)	1 (least deprived)	60 (20.1)	36 (23.8)	30 (23.3)	
	2	43 (14.4)	26 (17.2)	23 (17.8)	
	3	61 (20.5)	37 (24.5)	32 (24.8)	
	4	66 (22.1)	35 (23.2)	31 (24.0)	
	5 (most deprived)	68 (22.8)	17 (11.3)	13 (10.0)	
Age at assessment (years), median (range)			4.63 (4.18–7.17)	4.64 (4.18–7.17)	
Full-scale IQ at assessment, mean (SD)			108.03 (17.00)	108.00 (16.60)	

GA, gestational age; PMA, post-menstrual age at scan; IMD, index of multiple deprivation; IQ, intelligence quotient.

### MRI data

#### MRI acquisition

Infants underwent MRI at TEA on a 3 T system (Philips Medical Systems, Best, The Netherlands) sited on the neonatal intensive care unit using an eight-channel phased-array head coil. A paediatrician experienced in MRI procedures supervised the care of the infant during the MRI scan. Pulse oximetry, temperature and electrocardiography data were monitored throughout the session. Silicone-based putty (President Putty, Coltene Whaledent, Mahwah, NJ, USA), as well as neonatal earmuffs (MiniMuffs, Natus Medical Inc., San Carlos, CA, USA), were used for ear protection. Oral chloral hydrate (25–50 mg kg^−1^) was administered to infants whose parents chose sedation for the procedure (87% of infants were sedated). Whole-brain functional MRI was performed using a T2* gradient echo-planar image acquisition (sequence parameters: TR = 1500 ms; TE = 45 ms; flip angle = 90°; field-of-view: 200 mm); matrix: 80 × 80 (voxel size: 2.5 × 2.5 × 4 mm), 256 volumes (total scan time = 6 min 24 s). High-resolution anatomical images were acquired with pulse sequence parameters: T_2_-weighted fast-spin echo imaging: TR = 8670 ms, TE = 160 ms, flip angle 90°, slice thickness 2 with 1 mm overlap, in-plane resolution 0.86 × 0.86 mm. Diffusion imaging data were acquired in the transverse plane in 32 non-collinear directions with the following parameters: TR = 8000 ms, TE = 49 ms, voxel size: 2 mm isotropic, *b* value: 750 s/mm^2^, sense factor of 2, one non-diffusion-weighted image, *b* = 0.

#### Functional MRI preprocessing

All images were visually inspected to detect and exclude those with visible motion artefacts. Functional images underwent single-subject independent component analysis using FSL MELODIC^55^ followed by FIX^56^ for automatic de-noising and artefact removal. Independent component analysis was performed following removal of the first six volumes (allowing for T1 equilibration), motion correction with MCFLIRT, high-pass filtering (125s cutoff, 0.008 Hz) and automatic dimensionality estimation. No slice timing correction or spatial smoothing was applied at this stage. The standard FIX processing steps were modified to allow for standard-space masking using a population-specific neonatal template with tissue priors.^57^ The FIX algorithm was trained on hand-classified fMRI datasets, collected on the same scanner, from a sample of 40 infants aged 28–44 weeks GA, including both low-motion and high-motion subjects (see Ball *et al*.^58^, for further details).

Components were automatically classified as signal or noise (as described in Ball *et al*.^58^), after which the unique variance of each noise component as well as the full variance of the motion parameters and derivatives were regressed out of the data.^59,60^ Standardised DVARS, a framewise data quality index,^61^ was calculated before and after applying FIX. DVARS was significantly reduced following FIX clean-up [*t*(315) = 9.01, *P* < 0.001]. Finally, datasets with more than two standard deviations above the mean number of volumes detected as corrupted, as implemented by FSL Motion Outliers (calculated from DVARS), were removed, resulting in a final sample of 298 infants, of whom 129 (who had complete behavioural follow-up data) were included in further analysis.

Cleaned functional images from the remaining sample were resampled to 2 mm isotropic voxels and registered to a study-specific T_2_-weighted template using boundary-based registration. The template was generated from a subset of 161 participants using advanced normalization tools software as described in Lautarescu *et al*. ^62^. Data were spatially smoothed with a 4 mm full-width half-maximum Gaussian kernel.

#### Seed-based connectivity

For each participant, the mean raw signal timeseries were extracted from the left and right amygdala, respectively, as defined by the neonatal automated anatomical labelling (AAL) atlas.^63,64^ First-level general linear models were constructed using FSL FEAT,^65^ separately for the left and right amygdala, entering the mean seed timeseries as a regressor. As we were interested in localized effects of amygdalar connectivity relative to the whole-brain signal, global signal regression (GSR) was applied by adding mean whole-brain timeseries as an additional covariate. Although the choice of GSR is dependent on context and research question,^66^ it has been shown to strengthen associations between resting-state connectivity and behaviour^67^ and is likely to enhance subtle or regionally specific effects.^68^

#### Diffusion-weighted image processing

Diffusion-weighted images were preprocessed with FSL and analysed using tract-specific analysis, as described in Pecheva *et al*.^69^ and Kanel *et al*.^50^ Briefly, tract-specific analysis creates skeleton models of individual white matter tracts onto which diffusion data can be projected for statistical analysis. All subjects were registered to a study-specific template using a tensor-based algorithm.^70^ Following registration, tracts of interest were delineated from the template using deterministic tractography based on the FACT approach.^71^ Whole-brain tractography was seeded from a white matter mask and regions of interest were drawn manually according to the protocol described previously.^72^ Fractional anisotropy values were calculated for the UF bilaterally.

### Neurodevelopmental outcomes

Participants completed the Wechsler Preschool and Primary Scale of Intelligence (WPPSI-IV)^73^ to estimate their full-scale IQ, and a facial emotion recognition task developed in-house (described in detail in Kanel *et al*.^50^). In short, this task used static stimuli from the Dartmouth database of children’s faces,^74^ consisting of four boys and four girls displaying six emotions (happiness, surprise, fear, anger, disgust and sadness) and neutral expressions. Each emotion had two levels of intensity: either 100% (the original) or 50% (a morphed image of the emotional face with a neutral face). Children were asked to correctly determine which emotion each image was representing and the total number of correct responses were added up to create a total emotion recognition score.

The following parental behavioural questionnaires were administered: the Strengths and Difficulties Questionnaire (SDQ),^75^ measuring general childhood psychopathology (25 items categorized into five subscales: Emotional Symptoms, Conduct Problems, Hyperactivity/Inattention, Peer Relationship Problems and Prosocial Behaviour); the Children’s Behaviour Questionnaire—Very Short Form (CBQ-VSF),^76^ assessing children’s temperament, summarized into three broad scales (Negative Affectivity, Effortful Control and Surgency); the Empathy Questionnaire (EmQue),^77^ measuring empathy-related behaviours, summarized into three scales: Emotion Contagion, Attention to Others’ Emotions and Prosocial Actions and the Social Responsiveness Scale Second Edition (SRS-2),^78^ assessing social impairments associated with autism-spectrum behaviours, which provides subscales for social communication/interaction (SCI) and restricted interests and repetitive behaviour.

### Statistical analysis

Statistical analyses were performed in R Core^79^ and FSL FEAT. Factor analyses included data on 151 participants with complete neurodevelopmental data, using the following socio-emotional outcome variables: four SDQ subscales (Emotional Symptoms, Conduct Problems, Peer Relationship Problems and Prosocial Behaviour), three CBQ subscales (Negative Affectivity, Effortful Control and Surgency), three EmQue subscales (Emotion Contagion, Attention to Others’ Emotions and Prosocial Actions), the SRS-2 SCI subscale and accuracy on the emotion recognition task. The resulting three factors (*Emotion Moderation*, *Social Function* and *Empathy*) were used in subsequent analyses (see Kanel *et al*.^50^).

For each factor, two general linear models were built (for left and right amygdala, separately), probing the association between whole-brain amygdalar rs-FC and each socio-emotional factor controlling for sex, neonatal sickness index, PMA at scan and socioeconomic status (i.e. IMD) [as maternal age at leaving education and IMD were correlated (*r* = −0.15, *P* = 0.05), we chose IMD as a measure of social risk]. *Z*-scores were used for all continuous variables. Whole-brain activation was determined by a voxelwise *z*-threshold of 3.1 and a cluster significance threshold of *P* = 0.05 (whole-brain family-wise error corrected). Clusters were labelled according to the AAL atlas.^63,64^

Where a significant association was found between a socio-emotional factor score and amygdalar rs-FC, *post hoc* analyses were carried out to investigate associations between cluster-specific connectivity (i.e. mean extracted Beta values from the significant clusters) and individual variables contributing to the relevant socio-emotional factor score. All analyses were repeated after removal of outliers in terms of both behavioural outcomes and Beta rs-fMRI values, defined as values more than 1.5 times the value of the interquartile range beyond the quartiles. A Bonferroni-corrected significance threshold of *P* = 0.05/6 = 0.008 (accounting for two lateralities and three outcome factors) was used for all follow-up analyses.

Finally, due to previous findings showing an association between neonatal fractional anisotropy in the right UF and childhood *Emotion Moderation* scores in the same participant sample,^50^ structure–function associations were explored by calculating the Pearson correlation coefficient between mean fractional anisotropy of the UF and amygdalar rs-FC Beta values from specific clusters spatially located in grey matter regions connected to the UF.^80^

### Data availability

The data that support the findings of this study, including socio-emotional factor scores and extracted Beta values for significant clusters, are openly available at https://github.com/danakanel.

## Results

### Socio-emotional factors

As previously reported, factor analyses conducted on socio-emotional outcome variables revealed a three-factor structure: *Emotion moderation*, *Social Function and Empathy.*^50^  *Emotion Moderation* had positive loadings for CBQ-VSF Negative Affectivity and CBQ-VSF Effortful Control scores; *Social Function* included positive loadings for higher SDQ Emotional Symptoms, SDQ Conduct Problems, SDQ Peer Relationship Problems scores and SRS-2 SCI; as well as negative loadings for SDQ Prosocial Behaviour, EmQue Prosocial Actions and CBQ-VSF Surgency and *Empathy* had positive loadings for EmQue Emotion Contagion and EmQue Attention to Others’ Emotions scores. Emotion recognition scores did not substantially load onto any of the factors. A high *Emotion Moderation* score indicates a more negative affect, as well as a stronger ability to effortfully control emotions. A high *Social Function* score indicates more socializing difficulties and a high score for *Empathy* indicates more displays of empathy in the child.

### Association between neonatal amygdalar connectivity and socio-emotional factors

#### Emotion moderation

**Figure 1:**
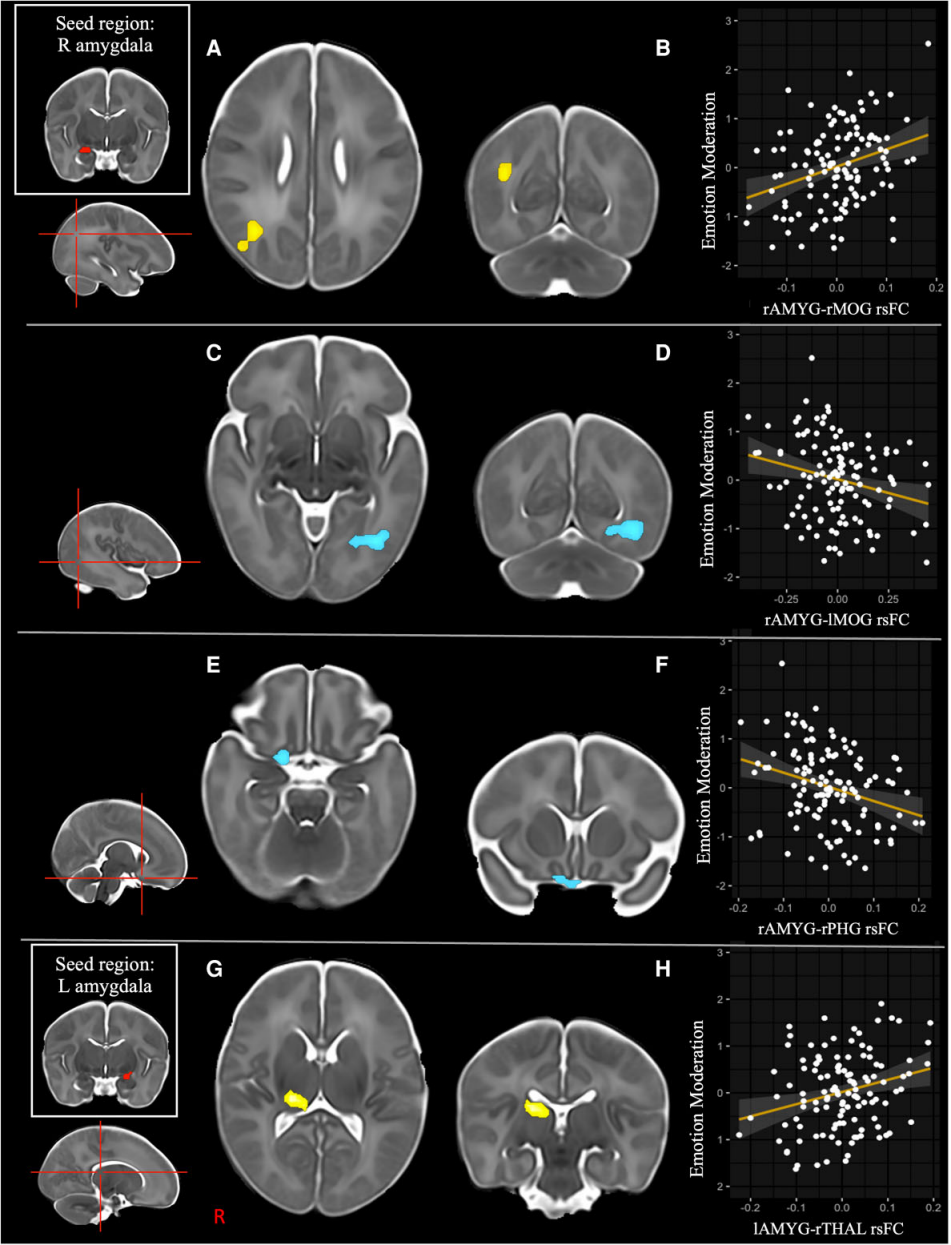
**Voxel-wise statistical maps and regression partial plots depicting associations between amygdalar rs-FC and *Emotion Moderation* scores.** Whole-brain voxel-wise statistical maps are family-wise error corrected. Right amygdala (rAMYG)–right middle occipital gyrus (rMOG): **A**. statistical map of rMOG cluster; **B**. association between rAMYG–rMOG and *Emotion Moderation* score. rAMYG–left middle occipital gyrus (lMOG): **C**. statistical map of lMOG cluster; **D**. association between rAMYG–lMOG and *Emotion Moderation* score. rAMYG–right parahippocampal gyrus (rPHG): **E**. statistical map of rPHG cluster; **F**. association between rAMYG–rPHG and *Emotion Moderation* score. Left amygdala (lAMYG)–right thalamus (rTHAL): **G**. statistical map of rTHAL cluster; **H**: association between lAMYG–rTHAL and *Emotion Moderation* score. All regression partial plots were created after outlier deletion. Yellow, positive associations; blue, negative associations; R, right; L, left. As images are not in MNI template space but rather in a neonatal template space, we have opted to use a crosshair to indicate exact peak position and the AAL labels to describe these regions.

Significant associations were identified between neonatal rs-FC of the right amygdala with three distinct clusters, depicted in whole-brain voxel-wise maps, and childhood *Emotion Moderation* scores (Fig. 1). Neonatal rs-FC of the right amygdala with a cluster with local maxima in the right middle occipital gyrus (MOG), extending to the right angular gyrus, was positively associated with *Emotion Moderation* scores (Fig. 1 Panel A). Neonatal rs-FC of the right amygdala with a cluster in the left MOG, extending to the left middle temporal gyrus and left lingual gyrus (Fig. 1 Panel B), and a cluster in the right parahippocampal gyrus (PHG) extending to the right OFC, the bilateral olfactory cortex, left gyrus rectus and right superior temporal pole, was negatively associated with *Emotion Moderation* scores (Fig. 1 Panel C and Table 2).

**Table 2:** Neonatal amygdala resting-state functional connectivity and childhood *Emotion Moderation* scores

Amygdalar seed laterality	Max Z	Location	Cluster size	Coverage	Association
**Right**	4.32	R middle occipital gyrus^a^	40	R angular gyrus, R middle occipital gyrus	Positive
**Right**	4.36	L middle occipital gyrus	58	L middle temporal gyrus, L lingual gyrus	Negative
**Right**	4.33	R parahippocampal gyrus^a^	55	B olfactory cortex, R orbitofrontal cortex, L gyrus rectus, R superior temporal pole	Negative
**Left**	4.27	R thalamus^a^	41		Positive

^a^ Results remained significant after Bonferroni correction and outlier deletion.

Seed: left or right amygdala. Max *Z*: Fisher’s *Z*-transformed correlation measure at cluster peak. Location: AAL area associated with cluster peak. Cluster size: number of voxels within cluster. Coverage: AAL areas included in cluster extent. Association: direction of association between rs-FC and ‘*Emotion Moderation*’ outcome. R, right; L, left; B, bilateral.

Positive associations were identified between neonatal rs-FC of the left amygdala with a cluster in the right thalamus and childhood *Emotion Moderation* scores (Fig. 1 Panel D and Table 2).

After outlier deletion and Bonferroni correction, associations between amygdalar rs-FC and *Emotion Moderation* scores remained significant for: right amygdala and right MOG (*n* = 120, *β* = 3.546, *P *= 0.001); right amygdala and right PHG (*n* = 123, *β* = −2.743, *P *= 0.003) and left amygdala and right thalamus (*n* = 122, *β* = 2.848, *P *= 0.003).

In order to aid interpretation of contributing variables driving the association between childhood *Emotion Moderation* scores and neonatal amygdalar rs-FC, we further analysed the two variables that meaningfully loaded onto the *Emotion Moderation* factor (CBQ-VSF Negative Affectivity and Effortful Control) separately and ran further regression analyses, adjusting for sex, neonatal sickness index, PMA and IMD (retaining a significance threshold of *P* = 0.008).

After correcting for multiple comparisons, all four clusters identified in the *Emotion Moderation* analysis were also significantly associated with Negative Affectivity scores; i.e. rs-FC of the right amygdala with the right MOG, left MOG and right PHG and rs-FC between the left amygdala and the right thalamus (Table 3). After removing outliers, all associations between amygdalar rs-FC and Negative Affectivity scores remained significant, except for right amygdala rs-FC with left MOG.

**Table 3:** Associations between Negative Affectivity scores and mean amygdalar rs-FC in significant clusters

Amygdalar seed	Resting-state functional connectivity cluster	Beta	*P*-value
Right	Right middle occipital gyrus	2.206	<0.001
Right	Left middle occipital gyrus	−1.442	<0.001
Right	Right parahippocampal gyrus	−2.623	<0.001
Left	Right thalamus	2.119	<0.001

All models adjusted for sex, PMA, neonatal sickness index and IMD.

All analyses significant after Bonferroni correction (adjusted *P*-value threshold = 0.008).

Only rs-FC of the right amygdala with the right PHG was significantly associated with Effortful Control scores, after controlling for multiple comparisons (Table 4). This association was no longer significant after outlier removal.

**Table 4:** Associations between Effortful Control scores and mean amygdalar rs-FC in significant clusters

Amygdalar seed	Resting-state functional connectivity cluster	Beta	*P*-value
Right	Right middle occipital gyrus	1.030	0.059
Right	Left middle occipital gyrus	−0.479	0.148
Right	Right parahippocampal gyrus	−1.836	0.003^a^
Left	Right thalamus	1.429	0.011

All models adjusted for sex, PMA, neonatal sickness index and IMD.

^a^ Analyses significant after Bonferroni correction (adjusted *P*-value threshold = 0.008).

#### Social function

No significant associations were found between *Social Function* scores and neonatal amygdalar rs-FC.

#### Empathy

No significant associations were found between *Empathy* scores and neonatal amygdalar rs-FC.

### Structure–function relationship

No significant correlations were found between participants’ fractional anisotropy values in the right UF and Beta values representing rs-FC between the right amygdala and right PHG (putatively connected to the amygdala via the UF)^80^ (*r_s_* = −0.1, *P* = 0.268).

## Discussion

The amygdalae are central to the brain’s emotional processing networks^29–32^ and investigating their functional connectivity early in life is critical for understanding the socio-emotional development of children who are vulnerable to affective disorders. Here we studied rs-FC of the amygdalae at TEA and childhood emotional outcomes following very preterm birth. We show that both stronger and weaker amygdalar rs-FC with cortical areas (MOG) and other subcortical regions that form the limbic system (PHG and thalamus) was associated with specific aspects of emotion regulation in middle childhood. As emotion regulation is potentially modifiable,^81^ establishing functional connectivity patterns to identify target groups for intervention has the potential to contribute to supporting very preterm children’s mental health.

### Emotion moderation

In this work, emotional development was summarized by a factor labelled ‘*Emotion Moderation*’, consisting of higher Negative Affectivity and Effortful Control scores. Negative Affectivity encompasses emotions such as anger, fear, anxiety, shame and disgust, and reflects a disposition to experience aversive affective states.^82^ Effortful Control refers to a self-regulatory temperamental trait which facilitates the modulation of reactivity by focusing attention or inhibiting/activating a behavioural response.^83,84^ Higher values reflect better Effortful Control ability. Although the combination of positive loadings of both Negative Affectivity and Effortful Control onto the *Emotion Moderation* factor may seem counterintuitive in the first instance, we have previously suggested that this factor may reflect an adaptive strategy, in that very preterm children could employ regulatory skills to moderate the impact of their reactive systems.^50^ Effortful Control has been suggested to act as a buffer against the development of psychiatric problems, by allowing individuals to use effective emotional responses to counter negative distortions or perceived threats.^85^ Indeed, children who score high on Effortful Control have been showed to have better social competence and Prosocial Behaviour, whereas those who score low tend to display negative emotionality,^86,87^ although findings from the literature have been inconsistent.^88,89^

We would like to propose an alternative interpretation to the *Emotion Moderation* construct. Early definitions of internalizing problems include difficulties based on overcontrolled symptoms that manifest when individuals attempt to maintain maladaptive control or regulation of internal emotional and cognitive states.^90,91^ Further, as part of Rothbart and Bates’ conceptualization of this trait,^92^ Effortful Control is formed by two regulatory processes: attentional control, or the ability to focus and shift attention^93^ and inhibitory control, or the ability to appropriately inhibit behaviour.^84^ These two processes should be considered separately when considering the role of Effortful Control in internalizing problems.^94^ Specifically, response inhibition has been positively associated with internalizing problems,^88,95,96^ possibly because what appears to be good inhibitory control may, in fact, reflect an overall inhibited, shy behaviour as a consequence of fear and anxiety.^97,98^ In their developmental model, Aksan and Kochanska^99^ posit that a fearful temperament in early childhood could facilitate the development of effortful inhibition in the future. Our study used the CBQ-VSF^76^ to measure Effortful Control, which focuses on complying to rules and exercising caution—typical of the cooperative and compliant shy child.^100^ Importantly, the combination of high Negative Affectivity *and* Effortful Control possibly due to an inhibited, shy personality resulting from fear and anxiety, may capture the behavioural profile of a typical preterm child: more internalized, less extroverted, shyer and more cautious,^101–105^ in line with the definition of ‘preterm phenotype’.^1,106^

### Neonatal amygdalar rs-fMRI and childhood *Emotion Moderation*

We found that *Emotion Moderation* scores in childhood were associated with neonatal rs-FC between the right amygdala and two regions: PHG (negative association) and right MOG (positive association). *Emotion Moderation* scores were also positively related to rs-FC between left amygdala and right thalamus. *Post hoc* analyses revealed these associations were mainly driven by Negative Affectivity scores.

The association of *Emotion Moderation* scores with rs-FC between right amygdala and a cluster with local maxima in PHG, and including OFC and temporal pole, is of particular interest given the importance of these regions in partially overlapping networks of the limbic system supporting emotion, memory^107,108^ and emotional memory.^109,110^ Functional connectivity between the amygdalae and PHG has also been studied as a predictor of emotion regulation in school-aged children.^111^ The OFC modulates the amygdalae’s response to external stimuli^112^ through inhibitory influences.^113^ Therefore, connectivity between the OFC and amygdalae is important in evaluating the affective significance of events.^114,115^ This regulatory mechanism may also apply to internal stimuli, as suggested by findings indicating an association between decreased amygdalae–OFC connectivity and increased anger,^116^ negative affect^117^ and anxiety.^118^

Decreased functional connectivity between the amygdalae and OFC^119^ and temporal pole^120,121^ has been reported in depression. Similarly, weaker amygdalae–PHG connectivity has been observed in individuals with depression^120,122,123^ and anxiety disorder.^124^ Taken together, our results suggest that rs-FC alterations in a network including amygdalae, PHG and OFC might represent an underlying biological mechanism linking very preterm birth and impairments in processes involving emotion regulation, and we speculate that this might explain very preterm individuals’ increased vulnerability to develop anxiety problems.^125^ Such altered rs-FC patterns in very preterm infants can already be observed at TEA and could be used as a connectivity fingerprint to predict later socio-emotional outcomes.

At a structural brain level, our findings are supported by diffusion MRI studies, which have shown an association between altered neonatal white matter microstructure in the right OFC and childhood socio-emotional problems.^126^ Further, the current results are in line with our previous work which assessed the relationship between neonatal diffusion characteristics of the UF and childhood *Emotion Moderation* scores.^50^ Anatomically, the UF connects cortical and subcortical regions including the amygdalae, PHG, OFC and temporal pole.^80^ Importantly, both Negative Affectivity and Effortful Control contributed to the association between *Emotion Moderation* scores and right amygdala–PHG connectivity, suggesting that it is indeed the combination of these two temperamental traits that is particularly sensitive to changes in early connectivity between these regions.

Connectivity between the right amygdala and right MOG, extending to angular gyrus, was positively associated with childhood *Emotion Moderation*, and in particular Negative Affectivity scores. Similar results were previously reported by Scheinost *et al*.,^46^ who showed that very preterm neonates exhibited stronger functional connectivity between the right amygdala and right occipital lobe compared with term-born controls. Of note, the angular gyrus is part of the default mode network, which has been implicated in affective regulation associated with anxiety and mood.^127^ Enhanced rs-FC between the amygdalae and several-default mode network brain regions has been observed in internalizing disorders,^128,129^ and has been further associated with altered self-referential thought processes and negative rumination.^128,130^ These findings could aid the interpretation of the observed association between right amygdala-angular gyrus rs-FC and Negative Affectivity scores in our sample.

Finally, we found a positive association between left amygdala–right thalamus rs-FC and childhood *Emotion Moderation* scores, with this association once again being driven primarily by Negative Affectivity scores. It has been postulated that sensory information is relayed through thalamic connections to the amygdalae for emotional appraisal,^131^ and animal studies have highlighted regulatory mechanisms of the thalamus on the amygdalae and the importance of this connection for negative emotions and memories.^132,133^ A direct connection between the amygdalae and thalami has also been identified in humans^134^ and altered connectivity between the two regions has been associated with social impairments and depressive symptoms in adolescents with autism.^135^ The interhemispheric pattern observed here (i.e. increased rs-FC between left amygdala and right thalamus and higher *Emotion Moderation* scores) is surprising; however, future research could elucidate these findings by considering previous observations of volumetric hemispheric asymmetries of both the amygdalae and the thalami following preterm birth.^45,136^

### Neonatal structural and functional associations

Our current and previous results^50^ suggest that both structural and functional connectivity between right amygdala and right PHG could be useful for gaining insight into typical and atypical socio-emotional development. However, when investigating the relationship between the two modalities, we did not observe a significant association between functional and structural connectivity of the right amygdala and right PHG at TEA, despite both being separately associated with later *Emotion Moderation* scores. Although the anatomical structure of the human cerebral cortex constrains function,^137^ structure–function couplings are not always evident and exhibit age-related changes.^138^ For example, the default mode network shows disproportionately large increases in structure–function coupling over childhood and young adulthood when compared with other functional systems.^138^ Further, whilst high level agreement of structure–function connectivity within the default mode network has been reported in adults,^139^ such clear associations are not observed in children, who despite exhibiting adult-like default mode network functional connectivity, display weak structural connectivity.^140^ Such age-dependent patterns of structure–function connectivity could explain our results.

## Limitations

A limitation to the current study is that amygdalar connectivity was only measured at one-time point at TEA. A recent study in term-born infants indicated that whilst some connections between the amygdalae and both subcortical (e.g. caudate nuclei, putamina and thalami) and limbic regions (e.g. hippocampi, parahippocampal gyri) were already present just after birth, some adult-like amygdalar rs-FC patterns (including connections with prefrontal and parietal cortices) developed over the first year of life.^42^ Future research in very preterm samples could further elucidate longitudinal changes in amygdalar rs-FC in the first few years of life. Another limitation is that our study did not include a control group, which limits the ability to draw conclusions as to the specificity of these results to very preterm cohorts.

Differences in IMD between the baseline sample, which showed a relatively even distribution between the five IMD quintiles and the final sample, with only 10% of participants belonging to the ‘most deprived’ IMD quintile, may also limit our findings. This suggests that those participants who were not followed-up in childhood were likely to be at higher social risk than those who were assessed.^141^

## Conclusions

The current rs-FC study complements our previous structural findings of a relationship between neonatal amygdalar connectivity and childhood emotional development. In particular, the important regulatory effects of specific brain regions (including the OFC, PHG and thalami) on the reactive amygdala are highlighted here. Communication within the limbic system and between the limbic system and the cortex is important for higher-order cognitive affective functions, such as emotion regulation, which has direct implications on psychiatric outcomes.^142–144^ Our results suggest that patterns of functional connectivity associated with later socio-emotional outcomes in very preterm children are already evident at the earliest stages of life. These findings could be used as a connectivity fingerprint to predict later socio-emotional outcomes, which in turn could inform preventative interventions aimed at averting and targeting emerging emotional disorders.

## Abbreviations

GA =: gestational age
IMD =: index of multiple deprivation
MOG =: middle occipital gyrus
OFC =: orbitofrontal cortex
PHG =: parahippocampal gyrus
PMA =: post-menstrual age
rs-FC =: resting-state functional connectivity
rs-fMRI =: resting-state fMRI
TEA =: term-equivalent age
UF =: uncinate fasciculus
